# Low invasive estrous synchronization protocol for wild animals: an example with melengestrol acetate in brown brocket deer *(Mazama gouazoubira)*

**DOI:** 10.1590/1984-3143-AR2020-0526

**Published:** 2021-01-06

**Authors:** Yuki Tanaka, Alice Pereira Americano, David Javier Galindo, José Maurício Barbanti Duarte

**Affiliations:** 1 Núcleo de Pesquisa e Conservação de Cervídeos (NUPECCE), Faculdade de Ciências Agrárias e Veterinárias, Universidade Estadual Paulista (UNESP), Jaboticabal, SP, Brasil

**Keywords:** Assisted reproductive technologies, estrus, neotropical deer, corpus luteum, fecal progesterone metabolites

## Abstract

Deer are sensitive to stressful stimuli by handling and their reproductive physiology could be altered by these procedures, making it necessary to develop less invasive protocols for ART. Melengestrol acetate (MGA), a synthetic progestin administered orally, appears as an alternative for estrous synchronization protocols (ESP), such as reported in cattle. Firstly, we compared two MGA doses (0.5 and 1.0 mg/day/animal), which would have suppression effect in estrous behavior (EB). Eight females were randomly and equally distributed in Group 1 (G1) and Group 2 (G2), which received 0.5 and 1.0 mg/day/animal respectively for 15 days (D1 to D15). Two cloprostenol (CP) applications were performed on D0 and D11. Estrus detection (ED) was performed every day. All females from G1 displayed estrus during treatment period, whereas all females from G2 displayed estrus after treatment, suggesting a suppressive effect of 1.0 mg in the EB. Once the suppressive MGA dose (1.0 mg) was defined, we used this dose for assessing ESP. The same eight females received 1.0 mg/animal for eight days (D-8 to D-1), followed by 0.25 mg of estradiol benzoate on D-8 and 265 μg of CP on D0. Feces for fecal progesterone metabolites (FPM) measurement were collected from D0 until seven days after the last day of estrus. Seven females displayed estrus between 12 and 72 h after CP application, which was followed by a significant increase in FPM levels (except female MG6), suggesting the formation of corpus luteum. After ED, females were placed with a fertile male to assess the fertility of the protocol. Pregnancy was confirmed by ultrasound 30 days after mating in 3/6 individuals. Although the low effectiveness of MGA protocol, it should be considered as a promising alternative in deer ESP since this protocol has less stressful effect on the animal during reproductive management when compared to other ESP.

## Introduction

The brown brocket deer (*Mazama gouazoubira*) is classified as “LC” (“Least Concern” – not immediately threatened) by the International Union for Conservation of Nature (IUCN) Red List of Threatened Species (Black-Decima and Vogliotti, 2016). It is considered a polyestrous and non-seasonal breeders and births have been observed throughout the year (Pinder, 1992; Zanetti et al., 2010), but birth peaks have also been described during mid-winter and wet season (Stallings, 1986; Bisbal, 1994). Furthermore, it is one of the most abundant deer species in the neotropical region, and the most common in Brazil. Due to the large number of individuals in captivity, it is considered an experimental model in studies involving reproductive biotechnology for other neotropical deer species that are at greater risk of extinction (Duarte, 1996; Munerato et al., 2008; Zanetti et al., 2014).

Reproductive management of deer species is based on protocols applied to domestic animals (Morrow et al., 2009) due to the similarity of their reproductive physiology with domestic ruminants. Thus, techniques involving artificial estrus manipulation are also similar to those developed for cattle and sheep (Asher et al., 2000). However, it is worth to note that basic reproductive aspects of each species should be considered for successful assisted reproductive technology (ART) applications (Asher et al., 1992; Comizzoli et al., 2000), since reproductive strategies vary broadly among deer species (Ungerfeld et al., 2020). In *M. gouazoubira,* data regarding basic reproductive aspects consist mainly in the definition of estrous cycle’s length (±26 days), the presence of post-partum estrus and length of ±208~215 days of pregnancy (Pereira et al., 2006).

Regarding ART, the intravaginal progesterone release device (CIDR^®^) has been used on several deer species from temperate (Morrow et al., 1992, 1995; Asher et al., 1992, 1993; Argo et al., 1994; Hosack et al., 1999) and tropical regions (Zanetti et al., 2010, 2014; Zanetti and Duarte, 2012; Cursino et al., 2014; Galindo et al., 2015). Nevertheless, CIDR-based estrous synchronization protocol in deer still requires physical manipulation to insert the device in the animal, which can cause stress in these species (Dobson and Smith, 2000; Morrow et al., 2009). This is the most important consideration regarding wild animals, which are more susceptible to stress compared to domestic ones. This is the case of cervids, mainly when it is also known that stressors associated with handling can result in the secretion of adrenal progesterone that can potentially interfere with reproductive parameters (Monfort et al., 1990).

In this context, oral synthetic progestogens, such as melengestrol acetate (MGA) can be a novel technique for estrous synchronization in deer species. MGA is commonly used in estrous synchronization of domestic ruminants (Patterson et al., 1989; Safranski et al., 1992; Jabbar et al., 1994; Powell et al., 1996) and its oral administration is the mainly advantage in the control of the estrous cycle. Besides, it is a cheaper and less risky alternative for these species. To date, MGA is used for contraceptive proposes in deer such as white-tailed deer (*Odocoileus virginianus*) to control population growth in zoos (Roughton, 1979; Raphael et al., 2003), but there are no reports of MGA applied to estrous synchronization protocol in deer.

For the above mentioned, this study aimed to a) determine the dose of MGA (1.0 versus 0.5 mg/animal/day) which inhibits the estrous behavior; and b) evaluate the effectiveness of MGA for estrous synchronization protocol in *M. gouazoubira*.

## Methods

### Animals and experimental design

In both Experiments 1 and 2, eight females (No. 8, weight 14.7–21.05 kg, age 3–11 years, nulliparous to pluriparous) were used and three males of the same species were used to detect estrous behavior. The animals belong to the Deer Research and Conservation Center (NUPECCE) from São Paulo State University/Jaboticabal-SP (20 ° S). All individuals were kept in individual pens (12m^2^) with olfactory and sound contact with conspecific females and males and were exposed to natural photoperiod fluctuation. They were fed with 0.5 kg of pelleted ration diet for horses (12% crude protein, 2% crude fat, 10% crude fiber, Purina Co., Paulinia, São Paulo, Brazil) provided once daily in the morning and approximately 1 kg/deer/day of perennial soybean *(Neonotonia wighti)* or mulberry branches *(Morus alba),* provided according to their availability in the field. Water was offered *ad libitum*. Experiment 1 was conducted from September to October 2016 and Experiment 2 was conducted from June to August 2017. This study was divided into two experiments, and in the first one, the adequate dose for inhibiting estrous behavior was defined. Once the dose was determined, it was used in the estrous synchronization protocol (Experiment 2).

### Experiment 1

#### Hormonal treatment

##### Melengestrol acetate

Melengestrol Acetate (MGA) ^®^ Premix (Pfizer) has 22 mg melengestrol acetate per 100 g excipient. According to the manufacturer, the recommended dose for cattle is 0.5 mg/animal/day, which corresponds to 2.28 g premix MGA. In this study, 0.5 mg (2.28 g) and 1.0 mg (4.56 g) MGA were tested. MGA administration was performed by mixing it with mashed banana, since banana is a palatable fruit to deer. The mixture was offered individually in the morning (8 and 10h). The complete intake was controlled to prevent residue from the mixture and environmental contamination.

##### Prostaglandin analog

**Figure 1:**
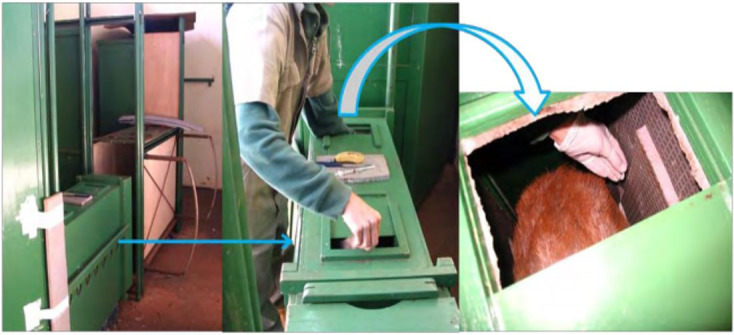
Adapted transport box used for sodium cloprostenol and estradiol benzoate application in the species *Mazama gouazoubira*. Reprinted from “Protocolos de superovulação em veado catingueiro (*Mazama gouazoubira*)” (doctoral thesis, p. 31), by ES Zanetti, 2009, Universidade Estadual Paulista, Faculdade de Ciências Agrárias e Veterinárias. Copyright 2009 by Eveline dos Santos Zanetti. Reprinted with permission.

Two intramuscular (i.m.) applications of 265 µg (1 mL) of sodium cloprostenol (PGF_2α_ analogue) (Ciosin; Schering Plow Coopers, Brazil) were administered at Day 0 and Day 11 for luteolysis induction (Zanetti et al., 2010). Thus, each female was conducted to an adapted transport box for deer species handling to guarantee an adequate control of the dose-volume during the hormonal application (Figure 1).

#### Division of groups

For Experiment 1, females were randomly and equally divided into two groups. Group 1 (G1) received 0.5 mg/animal/day and Group 2 (G2) received 1.0 mg/animal/day of premix MGA from Day 1 to Day 15.

#### Estrus detection

Signs of estrous behavior were determined by placing each female with a fertile male of the same species for 5 min twice a day between Day 0 and Day 20. Estrous behavior is defined as the period when females allow mating (Pereira et al., 2006), but in Exp 1 mating was not allowed.

### Experiment 2

#### Hormonal treatment

**Figure 2:**
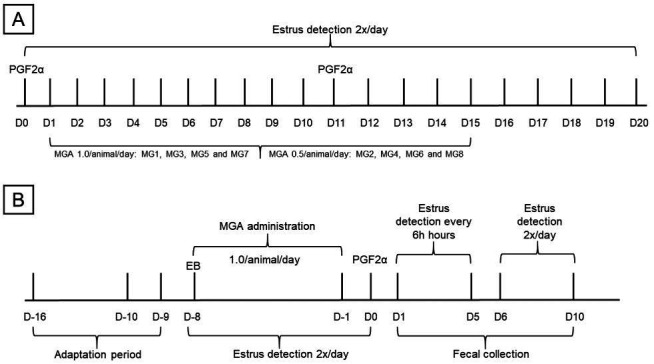
MGA-based treatments for experiments 1 and 2, respectively, in females of *Mazama gouazoubira*. (A) MGA treatment scheme for Groups 1 and 2. D1 and D11: application of sodium cloprostenol. Day 1 to Day 15: Group 1 received 0.5 mg/animal/day and Group 2 received 1.0 mg/animal/day. Day 1 to Day 20: detection of estrus 2 x per day. (B) Day – 16 to Day – 9: adaptation period. Day – 8 to Day – 1: administration of 1.0 mg/animal/day. Day – 8: administration of estradiol benzoate. Day – 8 to Day 0: estrus detection performed twice a day. Day 1 to Day 5: estrus detection period every 6 hours. Day 6 to Day 10: detection of estrus twice per day. Day 1 to Day 10: fecal collection.

From Day – 8 to Day – 1, females received 1.0 mg/day/animal of MGA mixed with mashed banana similar to Exp 1. Still on Day – 8, they received an i.m. application of 0.25 mg of estradiol benzoate (EB) (Sincrodiol®; Ourofino Saúde Animal Ltda., Brazil). They also received an i.m. application of 265 μg PGF_2α_ analogue on Day 0 (Figure 2). For cloprostenol and EB application, females were conducted to an adapted transport box for deer species handling.

#### Estrus detection

Estrus detection (ED) was performed as described in Exp 1 from Day -10 to Day 0. From Day 1 to Day 5, the ED interval was every 6 h after cloprostenol application and mating was allowed. The reduction in ED interval was performed to adjust the onset and the end of estrus. After this period, estrus was detected once a day for ten more days.

Since there are no reports of the use of MGA in estrous synchronization protocols in *M. gouazoubira*, ED was performed throughout the study period to verify the efficacy of this progestogen in estrous suppression.

### Fertility assessment and ultrasound (US) performance

To assess the fertility of the females treated with MGA, mating was allowed in all females that exhibited estrous behavior using three fertile males of the same species. Mating was allowed every six hours after ED. The US was performed 30 days after mating using the MyLab Vet30 Gold (Esaote, Italy) ultrasound equipment with a multifrequential linear transrectal transducer (7.5 MHz), coupled to an extender to allow its manipulation and equipment configurations were standardized. The females were chemically restrained with an association of i.m. xylazine hydrochloride (Rompun/Bayer, São Paulo, SP, Brazil; 1 mg/kg) and ketamine (Dopalen/Agribrands, Paulínia, SP, Brazil; 7 mg/kg).

### Fecal sampling

To assess the corpus luteum functionality after the synchronization protocol (Zanetti et al., 2010), fecal samples were collected to measure fecal progesterone metabolites (FPM). Samples were collected once a day/female at 8h to 10h from Day 0 up to seven days after the last day of estrous behavior manifestation. Samples were stored in plastic bags, correctly identified, and stored in a freezer at −20 ºC until processing.

### Fecal steroid extraction and enzyme immunoassay

Fecal steroid extraction was based according to methodology described in Tanaka et al. (2019). The validation of hormonal measurement was based on the methodologies described according to Brown et al. (2004), by observing the parallel arrangement between the standard curve and the curve formed by the pool of fecal extracts prepared by serial dilution and significant recovery of exogeneous progesterone (y = 1.1903x + 3.1282, R^2^ = 0.9943) added to fecal extracts. The interassay coefficients of variation for two separate internal controls were 4.8% (n = 7, 64% binding) and 4.2% (n = 7, 24% binding). Intraassay variations were < 10%. All results were expressed based on fecal dry weight.

### Statistical analysis

The duration of estrous behavior was estimated by direct observation in a six-hourly basis as described in the Exp 2. The corpus luteum formation was assessed by increasing FPM concentrations on the days following the last day of estrous behavior manifestation. The values of progesterone concentration of each female from the day after the last day of estrous behavior were considered basal and from these, the mean and standard deviation (SD) were calculated. Values greater than the criterion value (mean + 2SD) were considered indicative of the luteal phase (Thompson et al., 1998; Pereira et al., 2006; Polegato et al., 2018).

### Location and Ethical Statement

The research was conducted at Deer Research and Conservation Center (NUPECCE) from São Paulo State University/Jaboticabal-SP (20 ° S). Both Experiments 1 and 2 were approved by the Animal Ethics and Welfare Committee (Comitê de Ética e Bem-estar Animal, CEUA of the Faculty of Agrarian and Veterinary Science (Faculdade de Ciências Agrárias e Veterinárias, FCAV) UNESP, Jaboticabal, SP, Brazil (protocol number 18.335/16 and 009316/17).

## Results

### Experiment 1

#### Estrus detection

During treatment with MGA and after induction of corpus luteum regression with cloprostenol application, all females that received 0.5 mg/animal/day displayed estrus during treatment (Day 4 – MG8, Day 13 – MG6, Day 14 – MG2 and MG4). All females that received 1.0 mg/animal/day displayed estrus only after finishing MGA treatment (Day 17 – MG1, Day 18 – MG7, Day 19 – MG3, and Day 20 – MG5). Thus, 1.0 mg/animal/day was chosen for Experiment 2.

### Experiment 2

#### Estrus detection

During MGA treatment in the synchronization protocol, three females (MG4, MG5, and MG6) displayed estrus about two to three days after the EB application (Day – 8). Females MG2, MG4, MG6, and MG7 displayed estrus one day after the application of cloprostenol (Day 1), MG8 on Day 2, and MG1 and MG3 on Day 3. Female MG3 displayed estrus without successful mating. Female MG5 did not show estrous behavior in any moment after MGA treatment cessation and cloprostenol application.

### Ultrasound performance

**Figure 3:**
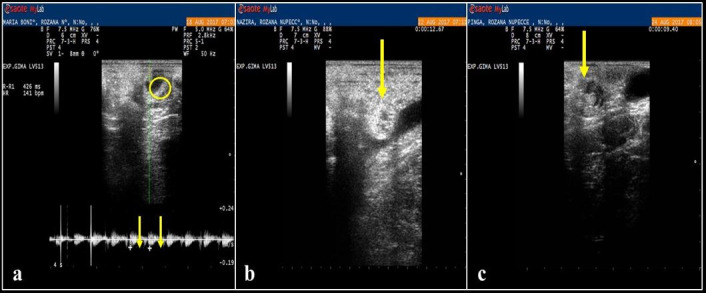
B-mode ultrasonographic evaluation of the uterus in females of *Mazama gouazoubira* with 30 days of gestation. a) Female MG1, yellow circle shows the embryo and yellow arrows indicate the heart rate of the embryo by Doppler ultrasound. b) Female MG4, yellow arrow indicates the gestational vesicle. c) Female MG8, yellow arrow indicates the gestational vesicle.

The pregnancy was confirmed in three females (MG1, MG4 and MG8 – Figure 3). The pregnancy diagnosis was negative for MG2, MG6 and MG7.

### FPM profile

**Figure 4:**
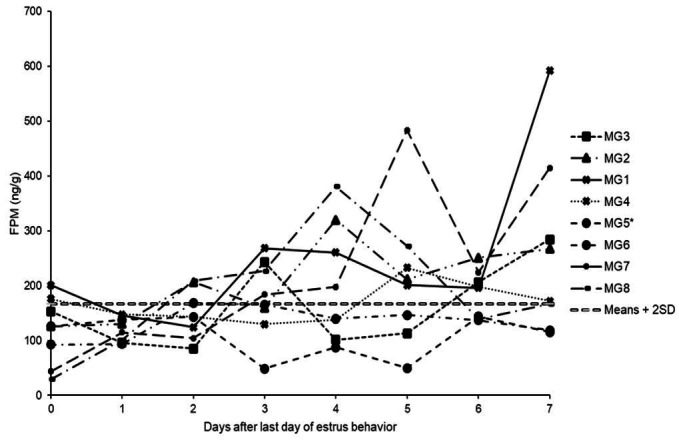
Fecal progesterone metabolites (FPM) levels profile in the eight females of *Mazama gouazoubira* from the last day of estrous behavior (Day 0) up to seven days. The increase in FPM was observed in Day 2 for MG2 and MG8, in Day 3 for MG1, MG3 and MG7, in Day 5 for MG4. Asterisk in MG5 indicates that this female did not show estrous behavior, but it is represented in the graph to show its FPM levels. The female MG6 showed estrous behavior but FPM levels did not along the days, indicating that this female failed to ovulate. The double dashed line indicates the considered limit of the means + 2SD above which indicates ovulation and corpus luteum formation.

FPM profile of each female is shown in Figure 4. The lowest concentrations of FPM were observed in the first days after the end of estrous behavior. The highest concentrations were observed from the third day after the last day of estrous behavior (except for females MG5 and MG6). From the FPM baseline, it was defined that ovulation occurred in females MG1, MG2, MG3, MG4, MG7, and MG8.

In total, seven females displayed estrus, but we observed that the corpus luteum possibly formed in six females (MG1, MG2, MG3, MG4, MG7, and MG8).

The increase in FPM concentrations occurred two days after the last day of estrus for MG2 and MG8, three days after for MG1, MG3, and MG7 and five days after for MG4. The female MG5 did not show estrous behavior and the FPM profile showed no evidence of corpus luteum formation, while MG6 showed estrous behavior, but did not show an increase in FPM levels in the days following the estrous behavior, suggesting anovulatory estrus.

### Onset and duration of estrous behavior

After interruption of MGA administration (Day -1) and cloprostenol application (Day 0), all females except MG5 displayed estrus between 12 and 72 hours, with an average of 35.14 h ± 8.33. The duration of estrus varied from 12 to 36 hours, with an average of 23.14 h ± 3.32. From Day 5 to Day 10, none of the females showed estrous behavior (Table 1).

**Table 1:** Estrus onset after cloprostenol application and estrus duration per individual in females of *Mazama gouazoubira* from experiment 2.

**Females**	**Time to onset of estrus (h)**	**Estrus duration (h)**
MG1	72	12
MG2	24	36
MG3	60	18
MG4	12	18
MG5	-	-
MG6	24	18
MG7	24	30
MG8	30	30
Mean ± SEM^*^	35.1±8.33	23.1±3.32

* Values are presented with mean followed by standard error.

## Discussion

Despite the small number of animals used in the present study, our results suggest a suppressive effect on estrous behavior in *M. gouazoubira,* when offering 1.0 mg/animal/day of MGA to females (Experiment 1), similar dose that has been used for contraception proposes in other deer species and members of the family Bovidae (Roughton 1979; Raphael et al., 2003; Patton et al., 2007). On the other hand, an MGA-based synchronization protocol with daily administration of 1.0 mg/animal/day (Experiment 2) did not demonstrate an efficient estrous synchrony, due to wide range at the time of onset of estrous behavior (12 to 72 hours). Estrous synchronization based in oral progestogen could imply in a less predictable estrous behavior timing, from the progesterone withdraw until the elimination of the active metabolites from blood (Martínez-Álvarez et al., 2007). A possible cause of this large dispersion in estrus onset display might be due to MGA metabolization, which can be stored in adipose tissue and be launched at different rates for each female after treatment cessation (Neff, 1983).

Additionally to MGA metabolization, another source of variation in estrous behavior onset could be explained by the single offered period of MGA administration, which could not provide a constant progesterone concentration in the blood (Windorski et al., 2008). According to Windorski et al. (2008), the repartition of the total dose of MGA in two periods (offered in the morning and evening) could result in a better estrous synchrony and ovulation rate in ewes, because this procedure can maintain progesterone in a high concentration in blood throughout the day (Kojima et al., 2000).

Despite these considerations, we emphasize that the total intake of the MGA mixture with mashed banana was rigidly controlled, offering it in an individual feeder to guarantee uniform consumption of MGA. Therefore, differently from the MGA offering in a non-individual feeder in domestic ruminant (Funston et al., 2002), the individual offering adopted in our study would assure more successful estrous synchronization outcomes and avoid residues in the feeder, since this contamination could act as an endocrine disruptor after the end of the synchronization protocol.

To suggest the occurrence of ovulation after MGA protocol, we have measured the FPM values from a day after the last day estrous behavior until seven following days in all females. A steady increase in FPM levels along these days would indicate the presence of the corpus luteum. In this view, our results might indicate that the corpus luteum was present in (6/8) females, which showed FPM values above the criterion value (mean+ 2SD). This increase in FPM can reach a three-fold value from the progesterone level observed during the luteal phase from the estrous cycle of the species (Pereira et al., 2006). Regarding these females that showed estrous behavior and allowed mating (6/8), three got positive to pregnancy diagnosis 30 days after mating, which might suggest that MGA based protocol did not compromised the viability of the oocyte. However, we highly recommend that further studies are needed to assess the quality of the oocyte and with a greater number of animals. It is worth to note that in wild animals, experimental sample sizes will always be small, which compromises the controlled and hypothesis driven research (Herrick, 2019).

Regarding the length of the MGA-induced estrus of the females of our study (12~36h, n=7/8) followed data already described in the literature, which ranged from 12-54.1h (n=5) (Santos et al., 2001), but shorter than in the Pereira’s et al. (2006) work (23~80.6h, n=10). We can also infer that the estrous behavior induced by MGA treatment was associated with nadir FPM concentration, which is consistent with the data described for *M. gouazoubira* (Pereira et al., 2006). This event is represented in feces after 12 to 24 hours (Schwarzenberger et al., 1996; Palme, 2005; Peter et al., 2018) and it is in agreement with nadir FPM values observed in all females of this study.

In a context of *ex situ* conservation programs management, it should be underlined that the MGA protocol is less stressful for animals than other types of synchronization protocols, such as CIDR-based synchronization protocol. The MGA protocol requires minimum contact with the animal and no chemical immobilization of female deer, avoiding the stress of anesthetic recovery. In some cases, chemical immobilization is required for CIDR insertion. Therefore, in the MGA protocol, the progestogen can be offered in a “friendly manner” mixed with a fruit, and the application of synthetic hormones such as estradiol benzoate and prostaglandin analogue can be administered by a dart (Duarte et al., 2010). In addition, MGA is economically more advantageous, since the cost per head is ($1/head) for MGA and ($12.79/unit, reusable for 4~5 times) for CIDR.

After the execution of this study, we have widely adopted the MGA based protocol in our reproductive management routine (e.g. offspring production of endangered species, artificial insemination procedures) and applied in other *Mazama* species such as *M. americana, M. nana* and *M. nemorivaga* (Quadros et al., 2020; Tanaka et al., 2020). The MGA protocol is a promising ART approach and this study brings new perspectives to *ex situ* conservation programs and it would probably have a significant impact on the conservation efforts of endangered deer species.

## Conclusion

Despite the variation in estrous synchrony, the authors propose the adoption of an MGA-based synchronization protocol as an ART approach by *ex situ* conservation programs in terms of welfare and animal risks. This protocol offers greater security for both the animal and the animal keeper, due to the minimum contact and handling required with the animal.
